# The Use of Social Capital in Teacher Research: A Necessary Clarification

**DOI:** 10.3389/fpsyg.2022.866571

**Published:** 2022-06-10

**Authors:** Thibault Coppe, Laura Thomas, Nataša Pantić, Dominik E. Froehlich, Marc Sarazin, Isabel Raemdonck

**Affiliations:** ^1^Faculty of Psychology and Educational Sciences, University of Louvain, Ottignies-Louvain-la-Neuve, Belgium; ^2^Department of Educational Studies, Ghent University, Ghent, Belgium; ^3^Moray House School of Education and Sport, The University of Edinburgh, Edinburgh, United Kingdom; ^4^Department of Education, University of Vienna, Vienna, Austria

**Keywords:** social capital, teacher education, teacher research, social network, professional development

## Abstract

In this paper, we present a critical reflection on the concept of social capital. We argue that there is no such idea of an umbrella concept of social capital. Instead, two overarching conceptualizations of social capital exist, namely individual social capital and collective social capital. As these conceptualizations of social capital are completely different, we emphasize that studies using social capital as a theoretical lens should clarify the concept as well as be consistent in the interpretation of the concept, from its definition to its methodological operationalization. In this article, we first map the two different conceptualizations of social capital. Next, these conceptualizations are illustrated with well-known teacher research studies, followed by examples of studies in which individual and collective social capital are mixed. Finally, we discuss the consequences of the use and the mix of these different conceptualizations in terms of measurement methods. Additionally, implications for teacher education are presented.

## Introduction

Throughout the past two decades, there has been a growing body of literature that recognizes the importance of teachers' interactions for their professional development (Moolenaar, 2012; Kyndt et al., 2016; Baker-Doyle and Yoon, 2020). Teachers' professional development has increasingly been considered as a “socially embedded” phenomenon. This, in turn, has brought the concept of social capital to the forefront of the domain of teacher research. A multitude of studies have highlighted social capital as a resource for teachers' learning (Daly et al., 2020), wellbeing and job satisfaction (Edinger and Edinger, 2018), support (Bristol and Shirrell, 2019), induction (Thomas et al., 2019; März and Kelchtermans, 2020), and turnover (Hopkins et al., 2019). In line with this growing trend, emphasizing the social side of teacher development has become mainstream in teacher research nowadays, making social capital a trendy concept (Baker-Doyle and Yoon, 2020).

But even though this increasing body of teacher research seems to build on the same fundamental concept of social capital, the concept is operationalized and interpreted in various ways. Scholars do not always explicate which conceptualization of social capital they are using and often mix different conceptualizations of social capital under the same “umbrella construct”.

In this paper, we argue that there is no such idea of an umbrella concept of “social capital”. Instead, two overarching conceptualizations of social capital exist, namely individual social capital and collective social capital. Individual social capital (also called network-based social capital; Bourdieu, 1980; Lin, 2001) represents a potential benefit for individuals that is embedded in social interactions (such as professional resources available through relations with colleagues). Collective social capital (also called civic capital; Putnam, 1994; Paccagnella and Sestito, 2014) represents a collective good shaped by the sum of individual behaviors and is rooted in the shared culture of a collectivity (such as schools' trust climate or schools' shared positive norms). As these conceptualizations of social capital are completely different, we emphasize that studies using social capital as a theoretical lens should clarify the concept (i.e., whether an individual or collective perspective on social capital is chosen) as well as be consistent in the interpretation of the concept, from its definition to its methodological operationalization. In the teacher research domain, many papers build on a definition of individual social capital while operationalizing the social capital construct with theories and variables related to both individual and collective interpretations of social capital. The most common example of this mix is to present teachers' social capital as a combination of the trust climate of their school and the resources that teachers can access through professional interactions with peers (see below for details). Such practices can be problematic as they may result in conceptual ambiguity (Son, 2020). Theoretically speaking, it makes the concept blurry, and methodologically speaking, it is problematic to measure one construct (e.g., individual social capital) with measurement methods related to another construct (e.g., collective social capital).

In this paper, we first map the two overarching different conceptualizations of social capital, namely individual social capital and collective social capital. Next, these conceptualizations are illustrated with well-known teacher research studies, followed by examples of studies in which individual and collective social capital are somehow mixed. Finally, we discuss the consequences of the use and the mix of these different conceptualizations in terms of measurement methods. As the teacher research domain is mainly rooted in the individual social capital conceptualization instead of the collective variant, attention is especially devoted to measurement methods for the former. The main contribution this paper seeks to make to the special issue is:

- to clarify the different definitions of social capital in their scientific historical context and to provide examples of them coming from the teacher research domain,
- to highlight that individual social capital is a concept often used in teacher research,
- to discuss the consequences of mixing different conceptualizations of social capital, and
- to explore the operationalization of individual social capital.

Our aim is not to write a systematic literature review on the concept of social capital (for systematic reviews see Portes, 1998; Tzanakis, 2013; Son, 2020; Demir, 2021), but rather to critically discuss the concept, especially its use in the domain of teacher research.

## Mapping the Different Conceptualizations of Social Capital

### Understanding the Concepts of Social Capital

While some sources attribute the first use of the term “social capital” to Hanifan or Weber (Claridge, 2004), others point to Durkheim and Marx as setting out its conceptual foundations (Portes, 1998). Through the years, social capital has been used in many disciplines (i.e., economy, sociology, psychology, education, etc. Dika and Singh, 2002), and with several different meanings (i.e., capital from a social point of view; resource of the community; social commonwealth; people's social condition, etc. Farr, 2004). Current uses of social capital build particularly on the works of Bourdieu (1980), Coleman (1988), Putnam (1994), Nahapiet and Ghoshal (1998), and Lin (2001). In this section, we will not focus on the origin of the term (see Farr, 2004 for more information) but will discuss its meanings on the basis of these five more recent conceptualizations. In particular, we will briefly highlight the different conceptualizations of social capital carried out by the works of Putnam, Bourdieu, Coleman, Nahapiet and Ghoshal, and Lin (see Table 1) as it will help us to show that there are mainly two overarching conceptualizations of social capital, namely individual and collective social capital (Son, 2020).

**Table 1:** Different conceptualizations of social capital (table inspired by Claridge, 2018).

	**Collective social capital (also called civic social capital)**	**Individual social capital (also called network-based social capital)**
Putnam (1994)	Social capital is a public good mostly referring to state-level social capital. It is built by citizen engagement in public affairs.	
Coleman (1988)	Social capital is resources available to actors in their network and shared norms that facilitate reciprocity.	
Nahapiet and Ghoshal (1998)	Social capital is resources available to actors in the network and shared norms and shared language that facilitate interactions.	
Bourdieu (1980)		Social capital is resources related to the possession of a durable network.
Lin (2001)		Social capital is resources embedded in one's social networks.

Putnam (1994) proposed a definition of social capital strongly rooted in the collective idea of social capital: “Unlike conventional capital, social capital is a public good, that is, it is not the private property of those who benefit from it” (p. 10). For Putnam, social capital refers to community-level goods such as trust climate or civic engagement (mainly at macro-levels such as the state level). This conceptualization of social capital has also been called “civic capital” (Guiso et al., 2011). As an example, in his work, Putnam showed the relation between state-level social capital and the educational performance of schools (Putnam, 2001). His concept of state-level social capital was the sum of individual “civic behaviors” such as engagement in social organizations, engagement in public affairs, engagement in volunteering communities, perception of social trust, and engagement in sociability. In his conceptualization of collective social capital, Putnam distinguished bonding social capital and bridging social capital (Gittell and Vidal, 1998). Bonding social capital refers to the community-level good among a homogeneous population (within-group) while bridging social capital refers to the community-level good shaped by combination of several populations (between-group) (Leonard, 2004). This distinction has inspired later work, even the work of those rooted in an individual social capital perspective.

Coleman (1988) defined social capital both as a public good and an individual benefit. Starting from the individual perspective, he defines social capital as resources available to actors. Illustrating individual social capital, he explains that if A has a social relation with B, he can ask B to do something for him or to give him advice. Therefore, B is a resource possessed by A because of a social bond between them. Adding a collective perspective on social capital, Coleman explains that this exchange between A and B only works if they trust each other to have reciprocal exchanges. For Coleman, in a larger social environment than this dyad, individual social capital only exists when there is a form of collective social capital, such as trust and norms of reciprocity. As such, he conceptualizes collective social capital as a condition for individual social capital. In other words, “norms and sanctions (i.e., a form of collective social capital) are a necessary condition for initiating and promoting social exchanges among actors (i.e., a form of individual social capital) in a community” (Son, 2020, p. 10). Coleman differentiates individual and collective social capital, suggesting that collective social capital can represent a favorable environment to individual social capital. The work of Coleman on social capital, however, has been highly criticized (Ponthieux, 2006; Tzanakis, 2013; Tlili and Obsiye, 2014): while the distinction between individual and collective social capital is visible in his work, he has never made this distinction clear, using the same social capital term to refer to individual and collective social capital (Son, 2020). In other words “Coleman obscures the notion of social capital by including under this term mechanisms that generate social capital (such as mutual expectation and group reinforcement of norms), the consequences of possessing it (such as privileged access to information), and the ‘appropriable' social organization that provides the context for the former two (sources and effects)” (Marrero, 2006, p. 5).

Close to the work of Coleman, Nahapiet and Ghoshal (1998) defined social capital as “the sum of the actual and potential resources embedded within, available through, and derived from the network of relationships possessed by an individual or social unit” (p. 243). While this definition seems to refer mainly to individual social capital, Nahapiet and Ghoshal present social capital as shaped by three dimensions that refer both to individual and collective perspectives of social capital: structural, relational, and cognitive. The structural dimension refers to the presence or absence of ties between individuals (equivalent to individual social capital). The relational dimension refers to trust between individuals and norms and sanctions as group guidelines (similar to collective social capital). Finally, Nahapiet and Ghoshal (1998) present the cognitive dimension as an addition to previous conceptualizations of social capital, referring to shared perceptions that facilitate interactions such as shared language and codes (also similar to collective social capital). These dimensions refer to the relationships and the structural features of social capital (Froehlich et al., 2020b). Parallel to Coleman, Nahapiet and Ghoshal have been criticized for mixing different concepts into the same notion. Fine (2010) wrote: “Nahapiet and Ghoshal ‘throw everything from their field into social capital', including a good dose of Bourdieu” (p. 219).

In contrast to Putnam's collective view of social capital, for Bourdieu (1980), social capital is mainly an individual good that exists because of membership within a group. He defined social capital as the resources (existing or potential) that are related to the possession of a durable relational network (Bourdieu, 1980, p. 2). He used social capital to conceptualize social exchange dynamics within the ruling classes. For Bourdieu, society is clustered in communities and actors are positioned within these communities. According to their positions, they have access to different resources. As such, there are social inequalities regarding the clusters of actors and the positions of the actors within their clusters. Having more economic and cultural capital predisposes individuals to being members of different clusters and to having good structural positions within these clusters. It enables “advantageous locations in social space in the competition for the appropriation of available scarce resources” (Tzanakis, 2013).

Following Bourdieu's perspective, Lin (2001) also theorizes social capital from an individual perspective. He defines social capital as “resources embedded in one's social networks, resources that can be accessed or mobilized through ties in the networks” (Lin, 2008, p. 54). This conceptualization is also called network-based social capital (Lin, 2008). In Lin's conceptualization of social capital, trust or collective norms are explicitly not conceptualized as a form of social capital and social capital *is* individual social capital (Son, 2020).

**Figure 1:**
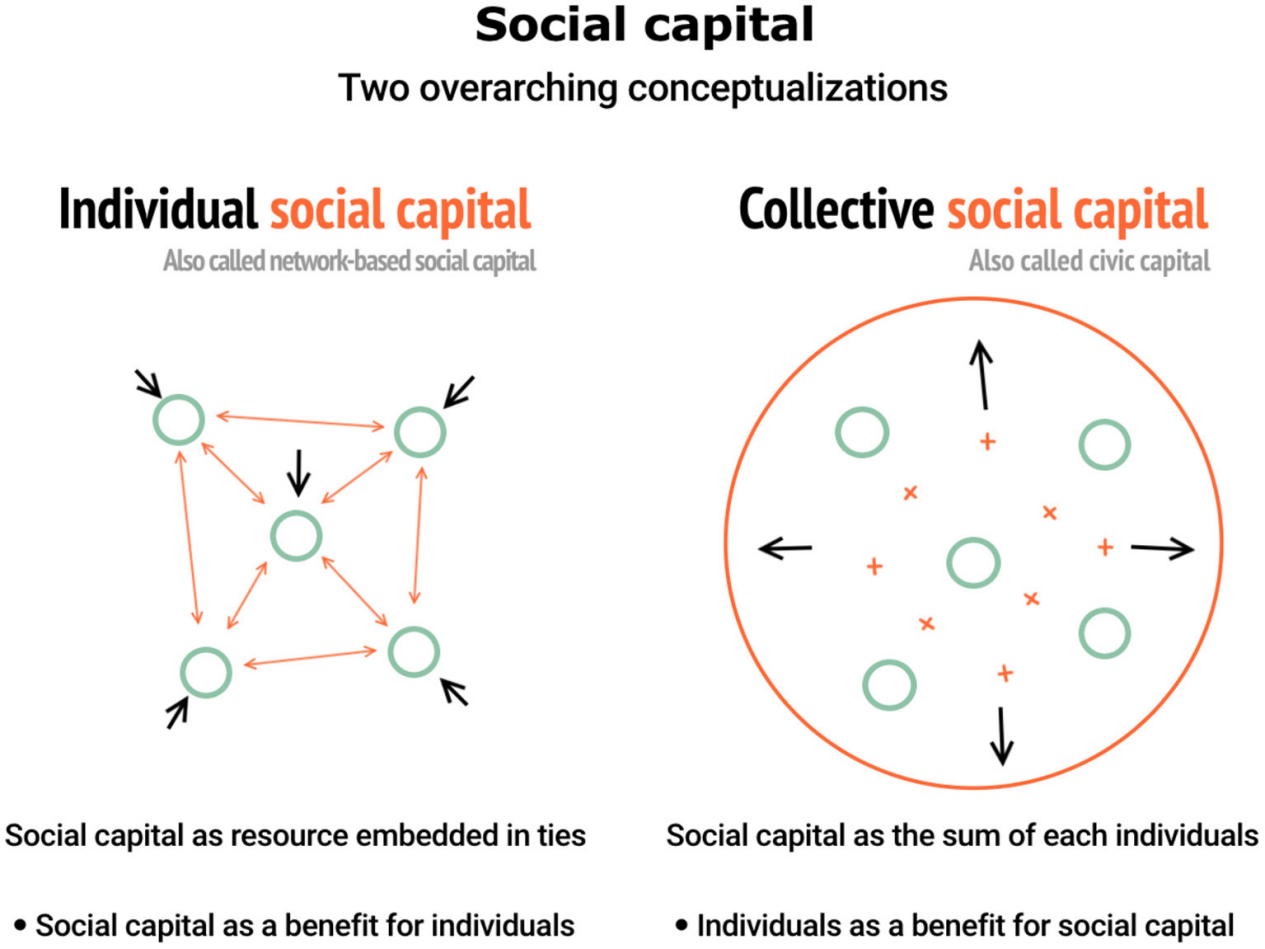
Two overarching conceptualizations of social capital (Coppe et al., 2021). In the individual social capital perspective, social capital are the resources that individual's access through their networks. In the collective social capital perspective, social capital is the common benefit (culture, norm,…) that is shaped by the sum of the individuals' behaviors.

Through the work of these authors, which represents the foundations of the two current overarching conceptualizations of social capital, we can conclude that individual social capital is the benefit that a person obtains as a function of their social position in a social network while collective social capital is the collective good that is shaped by a community (Godechot and Mariot, 2004). Individual social capital and collective social capital are sometimes linked, such as in Coleman's conceptualization, or completely distinct, such as in Lin's and Putnam's conceptualizations. Regardless of their possible link or opposition, these authors agree that an individual form and a collective form of social capital are not the same ideas (see Figure 1 for an illustration of these two conceptualizations). As we highlighted in this section, the work of Coleman and Nahapiet and Ghoshal has been criticized because their conceptualization of social capital tries to bring together two notions that are very different (Marrero, 2006; Fine, 2010). This duality represents a “considerable disagreement about whether social capital is a collective attribute of communities or societies, or whether its beneficial properties are associated with individuals and their social relationships” (Poortinga, 2006, p. 293). Though the disagreement in itself is not problematic, it can become a problem when “social capital” is used as an umbrella term without taking into account the debate regarding both conceptualizations. The risk here is that arguments drawing on collective and individual social capital are mixed, without (sufficient) knowledge about these two conceptualizations. In such cases, “conceptual chaos” can lead to the fall of the concept of social capital (Fine, 2010). Moreover, mixing individual and collective social capital can also pose problems with respect to research designs and operationalizations of social capital in empirical studies, which in turn may lead to incorrect conclusions.

### Social Capital in Teacher Research

Like other disciplines before it, the educational sciences have also embraced the concept of social capital for its theoretical value in understanding learning and development. This is especially visible in the teacher research domain. The increasing use of the social capital concept in teacher research follows the growing consensus among educational researchers that relationships matter (Moolenaar, 2012). In the past 15 years, empirical research on teacher development using the concept of social capital have flourished significantly (Demir, 2021). The concept of social capital is also more and more used as a part of other concepts mobilized in teacher research, such as the concepts of professional capital (shaped by human capital, social capital, and decisional capital: Hargreaves and Fullan, 2012) or intellectual capital (shaped by human capital and social capital, Daly et al., 2018). The underlying assumption of this trend is that social interactions are important sources of teachers' knowledge and professional development (Coburn and Russell, 2008; Baker-Doyle and Yoon, 2011). In teacher research, teachers' interactions and collegiality dynamics are increasingly described as resources for e.g., teacher retention, professional development, engagement, support, and learning (Daly, 2010; Thomas et al., 2019). The resources embedded in these social interactions have been naturally called teachers' social capital (Coburn and Russell, 2008; Baker-Doyle and Yoon, 2011). Until now, most of these studies have focused on teachers interacting with colleagues within their schools but some studies are going beyond and also look at the influence of teacher interactions between different schools (Cheah et al., 2011; Spillane et al., 2015).

Based on the idea that teachers' interactions are beneficial for teacher development, the social capital theoretical lens used in teacher research is strongly rooted in the conceptualization of individual social capital. This is also visible in the increasing use of the social network approach to study teacher development (Baker-Doyle and Yoon, 2020). As individual social capital refers to network-based social capital (Lin, 2008), the social network approach is particularly relevant for its operationalization.

Nevertheless, and this is where the concept becomes fuzzy, some studies in teacher research build their rationale on individual social capital, rooted in the idea that “relationships matter”, but theoretically and methodologically use a mixture of individual and collective social capital. Regarding this matter, we argue that even though teacher research becomes increasingly interested in the concept of social capital, its theoretical meaning has sometimes been overlooked. Suffering from its popularity, there is a risk that the concept becomes too general to be meaningful (Li, 2015).

To illustrate this problem, we now present examples of studies in the teacher research domain using strictly either individual social capital or collective social capital. The examples of studies using collective social capital are only illustrative. They do not represent the current trend in teacher research (which is mainly using an individual social capital approach) but illustrate how collective social capital can also be used in teacher research. We then present studies that mix both individual and collective social capital under the umbrella term of social capital, while being strongly rooted in the individual perspective of social capital. Following the criticisms made against Coleman and Nahapiet and Ghoshal in the past, we argue that the mix of these concepts can be conceptually and methodologically problematic. Table 2 presents the social capital conceptualizations of the examples presented in the next sections.

**Table 2:** Examples of studies using individual, collective, or a mixed form of social capital.

	**Conceptualizations**	**Operationalizations**
Spillane et al. (2012)	Individual	Social network approach
Struyve et al. (2016)	Individual	Social network approach
Bristol and Shirrell (2019)	Individual	Social network approach
Belfi et al. (2015)	Collective	Psychometric instrument
van Maele and van Houtte (2011)	Collective	Psychometric instrument
Moolenaar et al. (2014)	Mixed form with rationale related to individual social capital	Social network approach and psychometric instrument
Hopkins et al. (2019)	Mixed form with rationale related to individual social capital	Social network approach and psychometric instrument
Liou et al. (2017)	Mixed form with rationale related to individual social capital	Social network approach and psychometric instrument

#### Individual and Collective Social Capital in Teacher Research

As highlighted in the previous section, the individual social capital conceptualization in the context of teacher research could be synthesized as follows: *Teachers' relationships matter because they represent resources, as a form of capital, for teachers' development*.

An example of a well-known study in the teacher research domain using social capital from an individual perspective is the article of Spillane et al. (2012). In their study, social capital is defined as the advice and information available through relationships with colleagues. Their rationale for using the notion of social capital is that teachers can develop their knowledge “through on-the-job interactions with colleagues” (Spillane et al., 2012, p. 1118). Their study aimed to identify the factors that facilitate the development of teacher social capital. They used a quantitative social network approach (P^*^2 models, Van Duijn et al., 2004; Veenstra et al., 2007) to highlight factors associated with the occurrence of ties between staff members. Information about ties was obtained through the question “To whom do you turn in this school for advice or information about mathematics/reading/language arts/English instruction”. As such, the more a predictor was associated with the existence of ties between staff members, the more it contributed to teacher social capital.

Struyve et al. (2016) is another example of a study in the teacher research domain in which an individual social capital perspective is used. These authors analyzed how teacher social capital can reduce early career teacher attrition. They defined social capital as “the set of resources embedded in social relations” (Struyve et al., 2016, p. 200). They measured social capital as the number of colleagues with whom a teacher has interactions (instrumental interactions: “to whom do you go to for class-related information?”; or expressive interactions: “whom do you go to discuss more personal matters?”).

A lot of other studies could have been used as examples: Bristol and Shirrell (2019), Thomas et al. (2019), März and Kelchtermans (2020), and Coppe et al. (2021).

The use of collective social capital is way more uncommon in teacher research. An example of a study in the teacher research domain using social capital from a collective perspective is the article of Belfi et al. (2015). These authors use the concept of “school-based social capital” firstly developed by Goddard (2003). They measured collective social capital, at the school level, with scale items related mainly to the school's trust climate, commitment climate, and support climate. As another example of a study using collective social capital, van Maele and van Houtte (2011) analyzed to what extent homogeneity in teachers' beliefs about students' ability enhances school collective social capital. Their school collective social capital was measured through the trust in colleagues scale (Hoy and Tschannen-Moran, 2003).

#### Social Capital as a Mix of Individual and Collective Social Capital

While individual and collective social capital are two different concepts, a lot of studies in the teachers' research mix the two conceptualizations while in the meantime being rooted in the individual social capital perspective.

An example of a study in the teacher research domain mixing individual social capital and collective social capital under the umbrella term social capital is the study of Moolenaar et al. (2014). The article starts by stating that “Social capital theory explains how social relationships enable individuals to have access to and make use of, the resources that reside in their social networks” (Moolenaar et al., 2014, p. 208), referring to an individual perspective of social capital. Social capital, however, is later on in the article operationalized as having two components, namely relationships with colleagues and norms and values shared by group members. The first component is measured through a social network approach with the question “Whom do you turn to in order to discuss your work?” and for the second component a scale measuring trust in colleagues is used (Hoy and Tschannen-Moran, 2003). Moolenaar et al. (2014) hypothesize that social interactions create a trust climate in schools. Here, they describe trust climate as a factor of cooperation. One of their conclusions is that social interactions and trust climate may be in a circular relationship, one influencing the other. In other words, given the umbrella concept “social capital” used in this study, social capital predicts social capital, which is a tautological statement (Woolcock, 2010).

Another study mixing individual and collective social capital is reported in the article of Hopkins et al. (2019). Their study presents the importance of collaborations with colleagues for the retention of beginning teachers. In the article, adopting an individual approach, social capital is defined as “the resources embedded in social networks that can be accessed and used by actors for action” using the definition of Lin (2001, 2008). Then, social capital is seen as consisting of two dimensions, namely social network structure and relational trust (Hopkins et al., 2019). The social network dimension is measured with the question “to whom do you turn for advice or information related to curriculum, teaching, and/or student learning?” Trust is measured using the teacher-teacher trust scale (Tschannen-Moran and Hoy, 1998).

A third study mixing the conceptualization of social capital is the study of Liou et al. (2017). Here, social capital is defined as “the resources embedded in social networks that are formed by social relations” (Liou et al., 2017, p. 636) referring notably to the work of Lin about individual social capital. In the meantime social capital is described as shaped by two dimensions: social network structure and relational trust, referring to the work of Nahapiet and Ghoshal (1998). In addition, relational trust is defined as the “adhesive that connects individual actors” (Liou et al., 2017, p. 638) implying that one of the two dimensions of their social capital construct is an antecedent of the other.

We acknowledge that the studies listed in this section participated significantly to bring and to show the value of the social capital concept in the teacher research and they represent important contributions to the field. However, the approaches of each of these three studies seem to be rooted in Coleman's or Nahapiet and Ghoshal's view of social capital as they talk about social capital from an individual perspective and a collective perspective but in the meantime, anchor their definitions of social capital from an individual perspective. Beyond the idea that individual and collective social capital are sometimes considered as antagonist notions (Rostila, 2011)—inviting researchers to choose their playground well—we believe that mixing different conceptualizations of social capital, without making it clear, is problematic as it makes the concept blurry. Especially, when studies root their rationale in individual social capital (mainly developed in Lin's work), it can be problematic to operationalize part of the concept with variables related to collective social capital. From the individual social capital defender's point of view, as Lin wrote:

(…) trust has also been employed as a component or an indicator of social capital. However, its “social” nature is uncertain, and conceptually it might be more appropriate to consider it as an antecedent or effect rather than a component of social capital. (…) These discussions do not take away the conceptual significance of trust in its various forms (…). Rather, they remind us that it behooves us to refrain from equating trust with social capital. (Lin, 2008, p. 17).

Also, according to Son:

Trust is exogenous to (individual) social capital. Trust is an attitude toward people. (Individual) Social capital indicates the volume of instrumental and expressive resources within a network. In extreme cases, social capital can exist regardless of the degree or even the presence or absence of trust as long as there are operational ties. (...) If one understands social capital in a figurative and symbolic way, detached from the concrete resources commonly held by a network of people, one may call trust social capital. (...) Of course, a network-based theory of social capital refutes this idea because trust and norms are not resources in themselves. (Son, 2020, p. 149, 150)

And at the opposite, from the defenders of collective social capital, as Lochner et al. wrote:

Social capital is a feature of the social structure, not of the individual actors within the social structure: it is an ecologic characteristic. In this way social capital can be distinguished from the concepts of social networks and social support, which are attributes of individuals. (Lochner et al., 1999, p. 260)

Exchanges between teachers represent (individual) social capital as it represents a resource of advice, information, support, and learning. Some could also consider, in a symbolic way, that positive school climates (such as positive norms and trust) represent (collective) social capital as it shapes a positive environment to work and evolve. It is not so easy to believe that resources embedded in interactions and a positive school climate *are* the same notion that is called social capital. We do not argue here that one of the two notions is better than the other, but we believe it is necessary to choose one of the two research traditions to avoid a loss of conceptual meaning: “When social capital shifts from an individual-level relationship to a feature of a community, it becomes conceptually fuzzy” (Tzanakis, 2013). As a matter of fact, the teacher research domain has been more rooted in the individual perspective of social capital (Baker-Doyle, 2012), as this perspective represents precisely the idea that teachers' relationships matter for teachers' development. It does not mean that collective social capital is not something that could be interesting to study in schools. It does mean that scholars should be aware that building a rationale based on the idea that teachers' interactions represent resources for teachers' development is a rationale coming from the individual social capital conceptualization. Consequently, if scholars want to add the concept of collective social capital in their study, it should be carefully argued, and these two social capitals should be distinguished as two different concepts.

## Measurement in Teacher Research

As individual and collective social capital are two different concepts, it is not surprising to see that they are methodologically operationalized in very different ways (see Table 2, which illustrates this idea). In this section, we briefly present the most common measures of collective and individual social capital used in research on teachers. Then, we discuss how their mix can be problematic. Finally, we briefly discuss some current and future perspectives to measure individual social capital as teacher research is particularly rooted in this conception.

### Implications of Conceptualizations on Measurement

Collective social capital, originally theorized as a climate of civic engagement (Putnam, 2000), is mainly operationalized through a measure of school climate. This climate is sometimes considered as a trust climate among colleagues (van Maele and van Houtte, 2011), or a combination between different types of engagement: trust, commitment, and support between actors within the school (Goddard, 2003). Studies strictly using the concept of collective social capital in teacher research are mainly based on a quantitative design with psychometric surveys to measure collective social capital. We mainly noted the use of the teacher trust climate scale (Tschannen-Moran and Hoy, 1998; Hoy and Tschannen-Moran, 2003) or the school-based social capital scale (Goddard, 2003).

Individual social capital, conceptualized as the resources available through ties in one's network (Lin, 2001) is mainly operationalized with the social network approach. Numerous different social network methods have been used. We will come back later in this section to discuss these different methods. Rarely, individual social capital is measured with non-network scales with items related to support available from colleagues (e.g., Talis, 2018).

Unsurprisingly, studies mixing individual and collective social capital use both the social network approach and a psychometric instrument to measure their umbrella social capital. Mostly, the collective part of their social capital construct is measured with the trust in colleagues or derived scales (Hoy and Tschannen-Moran, 2003) and the individual part of their social capital construct is measured with *centrality measures*, which are social network data operationalizing to what extent an individual has access to others in their social network.

In the same way that collective and individual social capital do not have the same theoretical meaning, trust climate and number of ties with colleagues (which is a commonly used centrality measure: degree centrality) do not represent the same measurement object. Moreover, one can exist with or without the other. Some studies mixing collective and individual social capital analyzed the predictive link between trust and interactions with colleagues and concluded that one predicts the other (Moolenaar et al., 2014). This represents confusion between what *is* social capital and what are the antecedents or consequences of social capital (Tzanakis, 2013). As mentioned previously, it leads to the tautological statement: social capital predicts social capital (Woolcock, 2010). Following Lin and Son, we argue that for studies rooted in individual social capital but mixing individual and collective social capital, trust “is an antecedent or effect rather than a component of social capital” (Lin, 2008, p. 17) and as such, “trust is exogenous to social capital” (Son, 2020, p. 149). Subsequently, trust and interactions with peers cannot represent two parts of the same construct. Although they could represent two different constructs that are similarly named, namely individual and collective social capital but are clearly stated as different. In this way, they could be both present in the same empirical study, for example, by analyzing to what extent school trust climate (as collective social capital) predicts interactions between teachers (as individual social capital) or by testing this relationship in the opposite way since collective and individual social capital are expected to be in cyclic relationships (Moolenaar et al., 2014). In such an effort, there is no mix or risk of confusion between individual and collective social capital. Moreover, testing the relationships between individual and collective social capital properly would also allow to disentangle the effects of each concept.

Besides, we propose an analytical model that could allow to integrate both individual and collective social capital in the same study on the basis of a multilevel analytical framework. As collective social capital is often measured through psychometric scales (e.g., trust climate scale) and individual social capital is mainly measured through a social network approach (e.g., degree centrality), the model must take into account the non-independence of social network observations that is inherent to social network data (Tranmer et al., 2014). To this end, we inform the readers that the multiple membership multiple classification social network model (Tranmer et al., 2014; Coppe et al., 2022) is suitable for cross-sectional data and longitudinal data (implying to work with latent difference scores for longitudinal data) and the multilevel stochastic actor-oriented model is recommended for longitudinal data (Koskinen and Snijders, 2022). More information about these models is available in the above-mentioned references.

### Current and Future Prospects About Individual Social Capital Measurement Methods in Teacher Research

In this section, we focus on current and future prospects about individual social capital measurement methods in teacher research instead of discussing the prospects for both individual and collective perspectives because research in the teacher domain is mainly rooted in the individual perspective of social capital, as highlighted earlier in this paper.

Teacher individual social capital is mainly analyzed through the social network approach. It theorizes, represents, and analyzes nodes (actors) and ties (links) between actors within a social structure (the network). The social network approach is “a powerful analytic tool” to understand “the structure and content of teachers' professional relations” (Coburn and Russell, 2008, p. 226) and as such, it represents “an ideal framework for managing the complexity inherent in studying teachers' interactions” (Thomas et al., 2019, p. 134).

Through the social network approach, teacher individual social capital has been mainly measured with centrality measures, which are extracted from the network. Several centrality measures exist but mainly degree centrality (indegree centrality and outdegree centrality) and closeness centrality have been used as proxies for teacher individual social capital. Degree centrality is “the number of alters [other people in the network] that an ego [the focused person in the network] is directly connected to, possibly weighted by strength of tie” (Borgatti et al., 1998, p. 30). Closeness centrality is “the total graph theoretic distance from ego to all others in the network” (often inverted to keep a positive interpretation) (Borgatti et al., 1998, p. 31). Consequently, studies on teachers' individual social capital mainly follow a quantitative design based on analysis about antecedents, consequences, or comparisons in centrality measures (e.g., Struyve et al., 2016; Bristol and Shirrell, 2019; Coppe et al., 2022).

This prevalence of quantitative designs highlights an issue that is present more broadly in the literature on individual social capital. That is, most theories on social capital “exclusively accentuate the positive features of social capital while ignoring its possible downsides (...). Henceforth, theories on social capital should increasingly consider its possible dark side” (Rostila, 2011, p. 2). Indeed, using centrality measures to grasp social capital postulates that the more a teacher has interactions with colleagues, the more they possess social capital (Bristol and Shirrell, 2019). However, some interactions could be an obstacle for teachers' development. Consequently, beyond the use of centrality measures and quantitative designs to analyze teachers' social capital, its antecedent and consequences, it is important to go deeper into the meaning of these interactions. Combining qualitative data such as interviews to have more details about teachers' interactions seems a promising practice to this end (Penuel et al., 2009). This combination is in line with the emerging research tradition calling for mixed methods social network analysis (Froehlich et al., 2020a). Until now, only a few studies about teachers' social capital have used a mixed-method social network analysis approach (e.g., Thomas et al., 2019; Coppe et al., 2021).

## Conclusion

**Figure 2:**
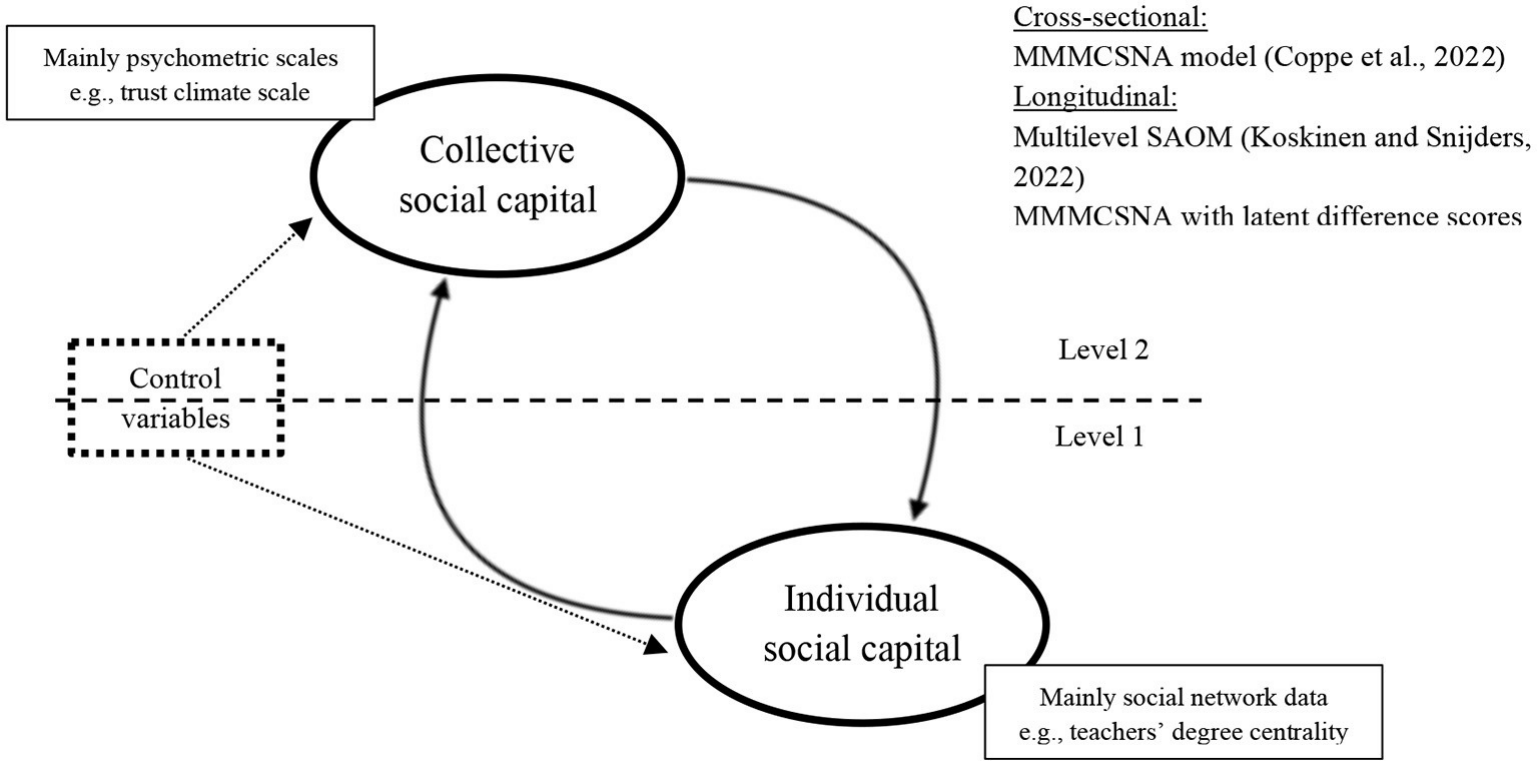
Analytical model for testing the relationship between individual and collective social capital. Outcome variable can be added. Individual or collective social capital would be then considered as mediator between collective/individual social capital and the outcome.

Considering the growing attention to the concept of “social capital” in studying teacher's professional development, the aim of this paper was to describe and illustrate the use of this concept in research on teachers. Different definitions and operationalizations are used and this makes “social capital” a blurry concept. Our study revealed that “social capital” is not an umbrella concept and that two main conceptualizations exist in the literature: individual and collective social capital. Up until now, the concept of individual social capital is mostly used in the teacher development literature. However, we found mismatches between definitions and operationalizations of the concept of social capital. A common practice is to use individual social capital as a definition while operationalizing the concept by using a mix of individual and collective social capital measures. This is problematic as it can lead to tautological statements, and as collective and individual social capital can exist both with and without each other. Using a mix of individual and collective social capital under the same umbrella concept of “social capital” has been criticized for a long time (Lin, 2008; Woolcock, 2010; Tzanakis, 2013; Son, 2020). Even those who look favorably upon the conceptual fuzziness of “social capital”—arguing that it has usefully “draw[n] attention to salient features of the social and political world”—argue that this “does not in any way absolve individual users [of the concept] of the requirement to be as precise as possible in articulating their particular definitions, theoretical moorings, and empirical referents” (Woolcock, 2010, p. 470–471). We, therefore, recommend that authors consistently explicate if they use individual and/or collective definitions of social capital, and align their definition with the way they operationalize the concept. In studies that measure both individual and collective social capital, we suggest that authors make the position of each of the main concepts in their theoretical model crystal clear. As suggested by Moolenaar et al. (2014), collective social capital might precede as well as follow from individual social capital, meaning that the two concepts are different. Moreover, it might also be interesting to examine possible interactions between individual and collective social capital, as we proposed in the Figure 2.

Concerning the measurement of individual social capital, we encourage researchers to measure not only the quantity of teachers' social interactions. Some interactions could have negative consequences, referring to the dark side of social capital (Rostila, 2011). We need to go beyond the quantity and grasp the quality and the meaning of interactions. A promising avenue to this end is to combine quantitative (quantitative social network approach) and qualitative (qualitative social network approach: Herz and Altissimo, 2021, interviews, and observation) research methods (i.e., mixed methods social network analysis). In this perspective, MMSNA is a promising area (Penuel et al., 2009).

Distinguishing individual and collective social capital and recognizing that individual social capital is at the forefront of teacher research also has implications for teacher education. As individual social capital can exist without collective social capital, it is important that teacher educators raise student teachers' awareness about the importance of building their networks in their future workplace and teach them about how to navigate the social structure of their school. Teacher induction research mainly emphasizes the importance of supportive school cultures for facilitating teacher induction and wellbeing. Nevertheless, novice teachers are agents, who can also actively build up their network and, as such, develop their individual social capital. “The early career teacher as a networker”, as written by Kelchtermans (2019), highlights the idea that teachers are not only passive receivers of the school structure (characterized by a positive or negative climate), but that they are also active developers of their individual social capital, through their agency. To conclude this point, we would like to cite März and Kelchtermans (2020): “(...) it is important to help early-career teachers to become more self-initiated and intentional in their networking (...). In order to be able to network, they need to understand how to navigate within the different networks, and they must be able to read the cultural and political scripts of their school's organization” (p. 9).

Even if we argue that individual capital can exist without collective social capital, it does not mean that collective social capital should be forgotten in teacher education. As proposed by Moolenaar et al. (2014) and illustrated in Figure 2, collective social capital can precede individual social capital. As such, raising the awareness of student teachers about the importance of building a trust climate and a sense of belonging (among other forms of collective social capital) is crucial. As such, while this paper mainly emphasized individual social capital in teacher research, we are also convinced that more research analyzing the conditions to foster schools' collective social capital would be valuable. Since collective development and organizational learning are becoming increasingly important for school development, studies on schools' collective social capital could nourish this field of research by emphasizing the organizational conditions that facilitate these collective dynamics.

Finally, in this paper, we did not address the distinction between bridging and bonding social capital—which was introduced mainly by Putman and then reused by individual social capital scholars. Nevertheless, as schools tend to be organized following departmentalized organizational logics, shaping disconnected subgroups of teachers (de Lima, 2007; Coppe et al., 2021), exploring the difference between within-group/department and between-groups/departments' social capital would be interesting.

## Author Contributions

TC: conceived the original idea, conceptualization, writing—original draft, and funding. LT: conceptualization, writing—original draft, and writing—review and editing. NP: conceptualization and writing—review and editing. DF: writing—review and editing and funding. MS: writing—review and editing. IR: writing—original draft (discussion section), writing—review and editing, funding, and supervision. All authors contributed to the article and approved the submitted version.

## Funding

This work was supported by the F.R.S.-FNRS: Belgian fund for scientific research. Grant Number: [F 6/40/5 - FRESH/FC 29830].

## Conflict of Interest

The authors declare that the research was conducted in the absence of any commercial or financial relationships that could be construed as a potential conflict of interest.

## Publisher's Note

All claims expressed in this article are solely those of the authors and do not necessarily represent those of their affiliated organizations, or those of the publisher, the editors and the reviewers. Any product that may be evaluated in this article, or claim that may be made by its manufacturer, is not guaranteed or endorsed by the publisher.
